# Efficacy and Safety of Intravenous Cladribine in Patients with Rapidly Evolving or Early Secondary Progressive Multiple Sclerosis

**DOI:** 10.7759/cureus.6995

**Published:** 2020-02-14

**Authors:** Foziah Alshamrani, Hind Alnajashi, Mohammed F Almuaigel

**Affiliations:** 1 Department of Neurology, College of Medicine, Imam Abdulrahman Bin Faisal University, Al Khobar, SAU; 2 Department of Medicine, King Abdulaziz University, Jeddah, SAU; 3 Department of Medical Education, College of Medicine, King Faisal University, Al-Ahsa, SAU

**Keywords:** cladribine, multiple sclerosis (ms), secondary progressive multiple sclerosis (spms)

## Abstract

Background

Multiple sclerosis (MS) is an autoimmune and demyelinating inflammatory disease that affects the central nervous system (CNS). The etiology of the disease remains unknown. Multiple theories highlight genetic, environmental, and infectious factors that may a role. MS is considered as the main cause of disability in young people. Cladribine, known chemically as (2-Chloro-2′-deoxyadenosine), is a purine analog chemotherapy used for hairy cell leukemia and other B-cell lymphomas. The goal of this study was to evaluate the safety and efficacy of cladribine in patients with rapidly evolving or early secondary progressive MS.

Methods

This observational, single-center, retrospective chart review at the MS Clinic in the Ottawa General Hospital, Ottawa, Canada. A total of 24 patients (median Expanded Disability Status Scale (EDSS) of 4.5) received cladribine (0.07 mg/kg/day) for four consecutive days every six months for ≥ 2 cycles with further cycles depending on lymphocyte recovery or disease activity to a maximum of eight cycles from 2005 until 2016 were included. Four patients who were already diagnosed with rapidly evolving or early secondary progressive multiple sclerosis (SPMS) were induced with cladribine. We evaluated relapse, EDSS, and magnetic resonance imaging (MRI) results.

Results

Out of 24 patients (ages ranging from 30 - 60), 80% were female. Median follow-up time was seven years. The mean relapse rate in the two years before patients were given cladribine was 1.25. Twenty patients had previously received multiple disease-modifying therapies (DMTs) (≥ 2) prior to receiving cladribine. Following cladribine, eight patients suffered 10 relapses (33.3% of the cohort). Annualized relapse rates (ARRs) were reduced from 1.25 to 0.42, which was statistically significant (p-value = 0.002). There was no mean difference in EDSS (p-value = 0.06): 16% deteriorated, 62% did not change, and 12.5% improved. New MRI activity (new T2 or Gad+ lesions) was noted in only seven of 24 patients.

Conclusion

Parenteral cladribine reduced the relapse rate from 1.25 to 0.42, which was statistically significant (p-value = 0.002). MRI activity in patients with rapidly evolving or early secondary progressive multiple sclerosis had a reasonable safety profile.

## Introduction

Cladribine is a purine analog chemotherapy used for hairy cell leukemia and other B-cell lymphomas known chemically as (2-Chloro-2′-deoxyadenosine) [1]. Cladribine halts cell replication and preferentially affects B and T lymphocytes due to an imbalance between activating and deactivating enzymes in these cell types. Cladribine is also believed to induce apoptosis or programmed cell death [2]. As a result, cladribine causes prolonged profound lymphopenia affecting both T-cells (CD4+, CD8+) and B-cells (CD19+) [3]. Parenteral cladribine has been approved for the treatment of malignancies, such as hairy-cell leukemia [1]. Cladribine has been used clinically for more than three decades in oncology populations [4]. It has also been investigated as a treatment for several autoimmune diseases, including rheumatoid arthritis (RA), systemic lupus erythematosus (SLE), and glomerulonephritis [5-6]. 

Cladribine has been previously evaluated in relapsing and progressive MS clinical trials and has shown promising results. In 1995, a double-blind placebo-controlled trial at the Scripps Research Institute showed a significant reduction in MRI activity which means that the lesions are inactive and it becomes more hypointense and disability progression in patients using parenteral cladribine at different doses compared to placebo. Serious side effects of cladribine include thrombocytopenia at higher doses, myelosuppression, and herpes zoster [7]. However, the potential for complications and availability of safer MS treatments such as interferons and glatiramer acetate may have caused a delay in further development and approval of cladribine for MS patients. 

Cladribine tablets were approved in August 2017 as a treatment for highly active relapsing MS. It was initially rejected in 2011 by European regulators and the US Food and Drug Administration because of safety concerns regarding the possibility of malignancy-risk [8]. Recent approval of cladribine tablets was based on efficacy shown in three Phase 3 clinical trials (CLARITY (NCT00213135), CLARITY Extension (NCT00641537), and ORACLE MS (NCT00725985)), in addition to over a decade of accumulated safety data from all MS patients exposed to cladribine in either parenteral or oral form (Premiere Registry, Premier, Inc., Charlotte, NC, USA), which clearly failed to demonstrate any substantial malignancy risk [9]. Post hoc analysis of CLARITY and CLARITY Extension showed up to 90% of patients were free of new lesions as demonstrated by MRI, and up to 81% of MS patients remained relapse-free four years after treatment with cladribine tablets, regardless of cumulative dose [10-11].

The most common side effect for patients taking cladribine tablets is an infection, particularly, herpetic infections. This is likely related to lymphocytopenia, which occurs in some patients.

Cladribine can be used either as parenteral or intravenous cladribine to treat highly active, relapsing-remitting MS patients (RRMS). Furthermore, we used parenteral cladribine for MS patients who presented with the significant breakthrough disease while on other disease-modifying treatments (DMTs) and were most likely transitioning to secondary progressive multiple sclerosis (SPMS). This study was completed before the market approval of oral cladribine. 

## Materials and methods

Data were analyzed for normality to determine whether to use parametric or non-parametric tests. Values are expressed as mean ± standard error of the mean or as median and were calculated using the Statistical Package for Social Sciences (SPSS) software, version 21 (IBM SPSS Statistics, Armonk, NY). Baseline Expanded Disability Status Scale (EDSS) and annualized relapse rate (ARR) were compared with post-treatment results using a univariate two-sample paired t-test. P-values less than 0.05 were considered significant at a 95% confidence level.

This investigation was a non-interventional/single-center, retrospective cohort, chart review conducted at the Multiple Sclerosis Clinic at the Ottawa General Hospital and Research Institute, Ottawa, Canada, from 2005 until 2016 and included 24 MS patients. Given the retrospective nature of the study and the use of anonymized patient data, all of which was consented to by the patients upon their entry to the MS clinic to use their information as needed for future research, requirements for ethical approval was waived. The research was according to the principle of the Helsinki declaration [9]. Patient inclusion criteria were as follows: age ≥ 18 years with relapsing-remitting MS (RRMS) and SPMS. Exclusion criteria were as follows: primary MS and being on experimental therapy. Twenty of these patients experienced a suboptimal treatment response to conventional DMT treatments, resulting in breakthrough disease. These patients had previously received at least one, or up to four, conventional DMT treatments prior to starting cladribine, including injectable interferon beta, glatiramer acetate, mitoxantrone, and natalizumab, from the time of their diagnosis with RRMS until the time of the disease transitioning to SPMS (median of 13 years). In addition, these patients showing signs of disease transition to SPMS were treated with intravenous (IV) cladribine (0.0875 mg/kg/day) for four consecutive days every six months for ≥ 2 cycles, with additional cycles depending on lymphocyte recovery or continued disease activity, to a maximum of eight cycles. Patients were followed every three months clinically and assessed neurologically (EDSS) in the MS and Hematology Clinics for any evidence of infection or other side effects for a median of seven years. Of note, three patients were unavailable after seven years. In addition, each patient in the study completed an MRI each year they were in the study.

Four of the study cohort patients presented with aggressive MS, which is defined as MS with multiple relapses in one year with an incomplete resolution, or those with an EDSS score of 4 within five years of the disease onset [10].These patients were given cladribine first as induction therapy before being prescribed any further DMT. We evaluated all patients in terms of the following standard outcomes: relapse rate, EDSS progression, and radiological evidence derived from brain MRIs for increased disease activity.

## Results

Twenty-four patients were included in this study. Median follow-up time post-treatment was seven years. Baseline clinical characteristics and demographic data are summarized in Table 1.

**Table 1:** Baseline Demographic and Clinical Characteristics EDSS - Expanded Disability Status Scale

Variable	Patients n=24
Age (median )	47 years (30 - 60)
Sex (Female %)	80%
EDSS (median)	4 (3.5 - 8)
Disease Duration (median)	13 years (8 - 33)

Twenty patients in the study received multiple or previous DMT (≥ 2) treatments prior to treatment with parenteral cladribine. Following cladribine treatment, 10 relapses occurred in eight (33.3%) patients. ARR was reduced from 1.25 to 0.42 (p = 0.002). EDSS showed a trend (p = 0.06): 16% of patients who showed continued deterioration, 62% stabilized, and 12.5% showed improvement. New MRI disease activity (new T2 or Gad+ lesions) was observed in seven of the 24 patients.

**Figure 1:**
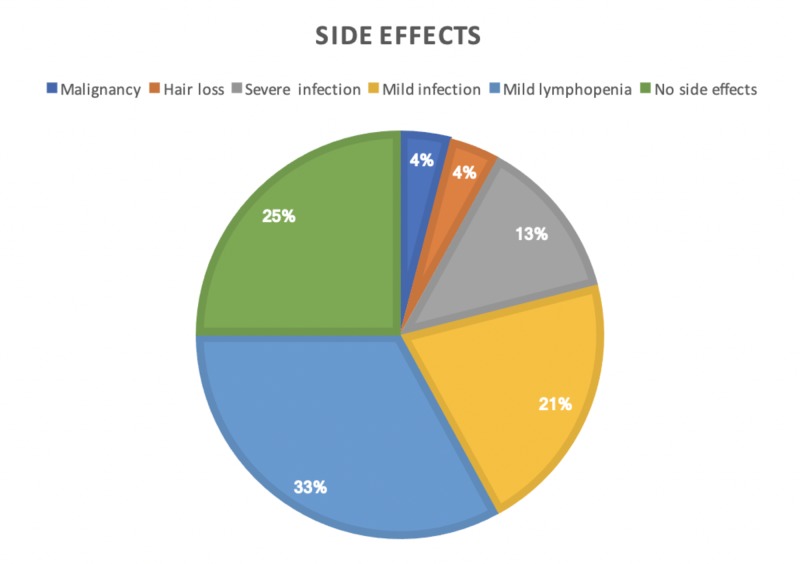
Side effects related to cladribine

Three severe systemic infections were reported, including two cases of severe pneumonia that required hospitalization and one case of severe urinary tract infection that led to the termination of medication. Moreover, five patients developed mild infections (urinary tract infection, upper respiratory tract infection). In addition, eight patients developed prolonged lymphopenia of Grade >3 for more than six months after cladribine infusion. Prolonged lymphopenia delayed further dosing. However, each of these patients recovered to Grade 0-1 in terms of lymphocyte counts before their next infusion. One patient developed Grade 4 lymphopenia but recovered after four months. Finally, one patient developed skin basal cell carcinoma and was promptly referred for surgical excision with no complication or recurrence. It is unclear if the case of basal cell carcinoma was related to parenteral cladribine administration. Safety and side effects data are summarized in Figure 1.

## Discussion

This single-center case series illustrated our experience using intravenous cladribine to treat RMS patients and early SPMS patients. Overall, cladribine has been shown to be effective for the treatment of early and late relapsing MS but is not commonly used for MS treatment, which is consistent with what has been reported in the clinical trial data [11]. Due to a good safety profile, cladribine is comparable to other chemotherapeutic agents used to treat MS, such as mitoxantrone. Mitoxantrone is associated with a significant risk of cardiotoxicity and leukemia and is used to treat relapsing patients with advanced disease, or for those who experienced a significant breakthrough while taking other DMTs, resulting in SPMS. 

In this study, cladribine showed reasonable efficacy in terms of relapse rate reduction and MRI activity. Furthermore, cladribine positively impacted Kurtzke’s EDSS. These results were similar to the efficacy and safety profile of the recently approved oral form of cladribine. The dosing regimen used in our study (0.0875 mg/kg/day for four days every six months) was parenterally equivalent to the approved tablet form in terms of absorption, distribution, and bioavailability [11]. Oral cladribine bioavailability is between 37% and 51%, with approximately 20% of cladribine bound to plasma proteins [12]. In addition, 25% of the dose is excreted in the urine and 3.8 ± 1.9% as a metabolite following oral dosing [13]. Cladribine was generally well-tolerated by SPMS patients and may be a suitable substitute for other chemotherapeutic agents, such as mitoxantrone, due to its superior safety profile, which is consistent with what has been reported in the clinical trial data.

Though oral cladribine is currently approved for the treatment of RRMS, this study supported the use of parenteral cladribine for the treatment of SPMS patients. Positive outcomes in this study suggest that parenteral cladribine could be used as a relatively inexpensive alternative MS treatment, particularly in poor populations with limited access to more expensive and riskier MS treatment options, such as mitoxantrone, which costs $429 USD in the United Kingdom per month and $908 USD in the United States per month [11]. 

In addition, other significant markers, including cerebrospinal fluid (CSF) neurofilament light chain (NFL) as an indicator for neuro-axonal damage, as well as the sensitive index of treatment effect, were studied [13]. Recently, Yildiz et al. found that cladribine may be an effective treatment option for individuals with non-relapsing deteriorating progressive multiple sclerosis as determined by the reduction of disease activity [14].

## Conclusions

This retrospective observational study with low patient numbers provides some evidence about the real-world tolerability of cladribine. However, some patients with aggressive MS may be resistant to cladribine clinically and radiologically, as some patients failed to show a reduction in lymphocytes counts. Therefore, future clinical studies should be conducted to evaluate parenteral cladribine treatment of secondary progressive MS with larger patient numbers.

Our observations showed that parenteral cladribine may be effective at reducing relapses, improving MRI activity, and slowing disease progression in patients with rapidly evolving MS. With limitations in SPMS treatment due to safety profile, in our opinion, parenteral cladribine is as safe as oral cladribine but has some limitations in which some patients refuse to take this medication due to the side effects. 
